# Microbial Primer: Transposon directed insertion site sequencing (TraDIS): A high throughput method for linking genotype to phenotype

**DOI:** 10.1099/mic.0.001385

**Published:** 2023-11-01

**Authors:** Isabel A. Warner, Weine J. Kok, Nicole Martinelli, Zihao Yang, Emily C. A. Goodall, Ian Henderson

**Affiliations:** ^1^​ Institute for Molecular Bioscience, University of Queensland, St Lucia, Australia

**Keywords:** bacteria, genetics, genotype, mutant, phenotype, TraDIS

## Abstract

Genetic screens are a key tool for linking phenotype and genotype. Transposon mutagenesis was one of the first genetic methodologies to associate genetic loci with phenotypes. The advent of next-generation sequencing transformed the use of this technique allowing rapid interrogation of whole genomes for genes that correlate with phenotype. One method is transposon directed insertion-site sequencing (TraDIS). Here we describe the method, recent developments in technology, and the advantages and disadvantages of this method compared to other genetic screening tools.

## Introduction

**Fig. 1.**
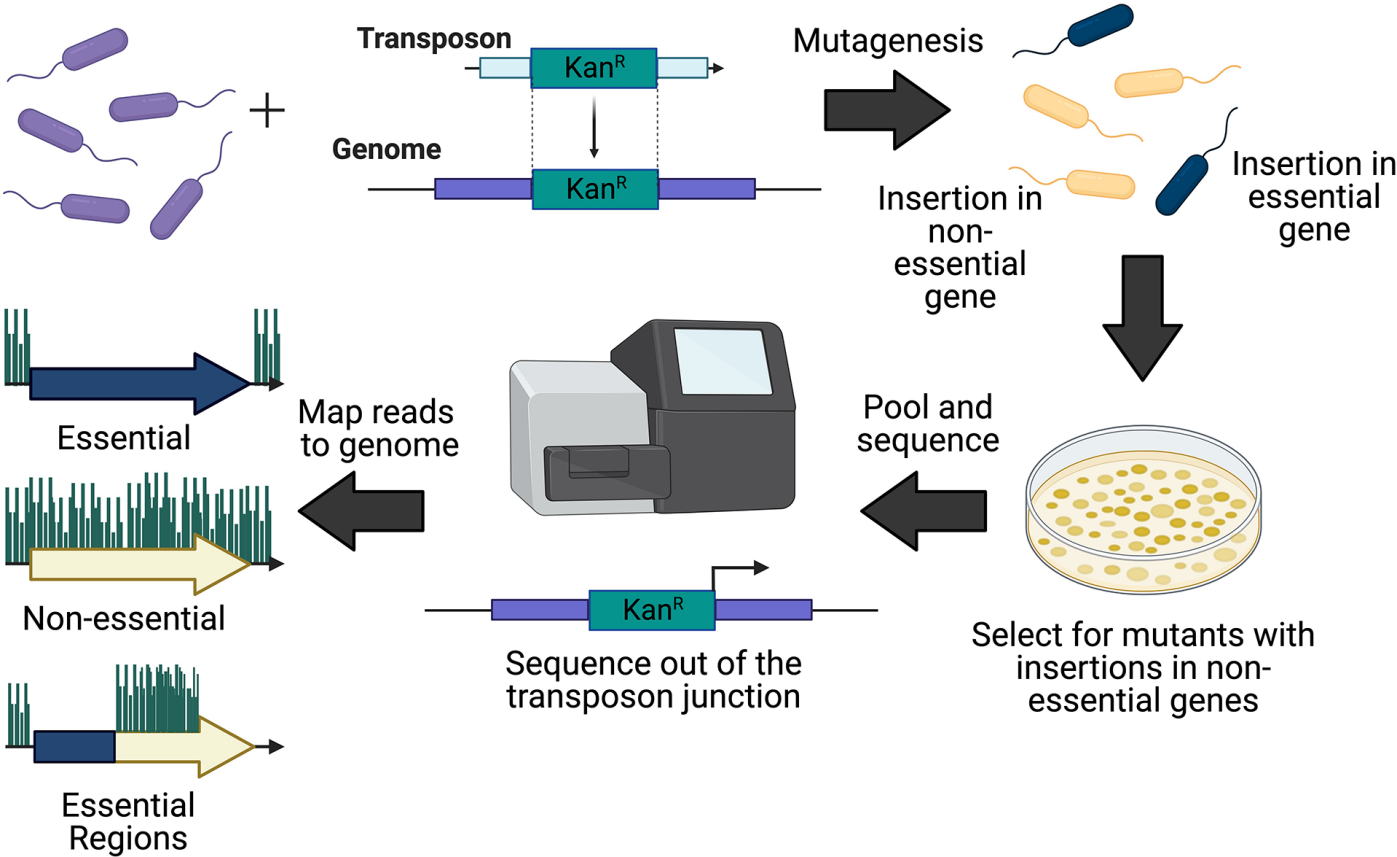
Overview of transposon insertion sequencing method. To construct a transposon mutant library, the bacterial strain of interest (top left) is transformed with the transposon containing a selectable marker (here shown as kanamycin resistance). The output mutant pool contains cells with mutations in essential genes (blue) and non-essential genes (yellow). Following growth on selective media to isolate successful transformants, mutants with insertions in essential genes will be non-viable, and not represented in the final mutant pool. Surviving mutants are collected, pooled, and prepared for sequencing. Sequencing is done from one end of the transposon outward into the genomic DNA. Bioinformatic analyses can then map this sequence back to the reference genome to determine the precise position of transposon insertion. This can then be visualized and quantified to identify essential genes, non-essential genes, and essential regions of genes or genomic sequences.

Genetic screens are a key tool for linking phenotype and genotype. They have been applied to study the fundamental biological processes that influence an organisms ability to grow and survive under a range of conditions, and the ability of bacteria to cause disease. Transposon mutagenesis, disruption of a chromosome by transposon insertion, is one of the earliest methods of genetic manipulation and screening. With the advancement of short-read whole-genome sequencing, four methods were developed to link these two technologies. Transposon sequencing (Tn-seq), insertion sequencing (INSeq), high throughput insertion tracking by deep sequencing (HITS), and transposon directed insertion-site sequencing (TraDIS) were developed as high throughput methods to rapidly identify previously unknown gene functions and regulatory networks. Collectively, these methods are termed transposon insertion sequencing (TIS) [1]. The focus of this primer, TraDIS, is a method that couples transposon mutagenesis with amplicon sequencing of the transposon-genomic DNA (gDNA) junction to identify the transposon insertion site. While there are differences in the specifics of each method (Table 1; compared in detail elsewhere [2]), all four methods share the same overarching approach. First, a mini-transposon, a piece of DNA containing a selectable marker flanked by inverted repeats that are recognized by a transposase enzyme, is introduced into the cell along with an exogenous transposase. Subsequently, the transposase mediates the random incorporation of the mini-transposon into the genome. Notably the mini-transposon lacks a gene encoding a transposase so that insertion can only occur once. Each incorporation produces a cell with a mutation that can be selected after growth on agar containing the appropriate selection conditions. These mutants are then pooled, gDNA is extracted and the transposon-gDNA junctions are isolated and sequenced to identify the location of the insertion. Importantly, this can be done at scale enabling the construction and sequencing of millions of mutants in tandem (Fig. 1).

**Table 1.** Transposon insertion site sequencing (TIS) methodologies

Method	Transposon type	Delivery	Fragmentation
Tn-seq	Mariner *HimarI*	Suicide plasmid, temperature sensitive plasmid	*Mme*I restriction
INSeq	Mariner *HimarI*	Suicide plasmid, temperature sensitive plasmid	*Mme*I restriction
HITS	Mariner *HimarI*	Suicide plasmid, temperature sensitive plasmid	Sonication
TraDIS	Mini-Tn*5*	Transposome complex, suicide plasmid, temperature sensitive plasmid	Sonication

The TIS methods differ in their choice of insertion element and preparation for sequencing. Briefly, there are two commonly used transposon-based systems: the *mariner HimarI* transposon, which inserts into TA dinucleotides, and the modified mini-Tn*5* transposon, which has a negligible bias for GC-rich regions. INSeq, Tn-seq and HITS use the *mariner* transposon. However, in the INSeq and Tn-seq methods the *mariner HimarI* transposon has been adapted to introduce a restriction site in the transposon inverted repeat region recognized by the *Mme*I restriction enzyme, which precisely cuts genomic DNA 16 bp up- and downstream of the insertion before amplification, sequencing, and mapping back to the reference genome. TraDIS and HITS shear the DNA prior to sequencing, which produces DNA of varying sizes, and can introduce bias during PCR, as smaller fragments are amplified first. TraDIS is the only method that uses the mini-Tn*5* transposon. Because the *mariner* transposon insertion site requires a specific TA dinucleotide, all possible insertion sites are known, which can be used to identify when the library has reached saturation. However, this transposon has limited ability to produce dense libraries in GC-rich genomes and lacks resolution in genomic regions with high GC content, including large numbers of core genes (shared amongst all strains). For this reason, it can be more difficult to reach high levels of transposon insertion saturation in all areas of the genome. This can create difficulty when determining the essentiality of short or GC-rich genes, particularly in low-density libraries where stochastic effects on the number of insertions in a gene can heavily confound essentiality classifications. The mini-Tn*5* transposon, by contrast, does not have this issue, and has been used to create libraries with insertion densities approaching codon level resolution [3]. The increased resolution greatly reduces mischaracterization of essential genes, but can also reveal additional genomic features such as promoter position and mis-annotated start codons [4]. The implications of transposon choice, and other parameters, on statistical analyses have been discussed extensively elsewhere [5].

## Applications of TraDIS

### Essential gene analysis

In its simplest form, TraDIS is used to identify the essential genes of an organism. Essential genes are required for an organism’s growth and survival, and result in its reproductive success. Because of their impact on survival, all known targets of bactericidal compounds are essential. Therefore, in addition to understanding basic bacterial biology, identification of essential genes is fundamentally important to identify targets for novel antimicrobials.

To identify essential genes, transposon mutants are pooled to create a mutant library. If the transposon inserts into an essential gene or region, the mutant will be non-viable, and not survive outgrowth selection following transformation. However, if the transposon inserts into a non-essential gene or region, the mutant will form a colony and be represented in the final library. The transposon-gDNA junctions of the mutant pool can then be sequenced *en masse* to identify regions of the chromosome that can be disrupted. With sufficient mutant density this method can identify all genetic loci that are essential for bacterial growth and survival (Fig. 1).

Prior to the introduction of high-throughput TIS methods, essential genes were identified by sequential targeting and deletion of each annotated gene. Failure to construct a mutant was an indication of gene essentiality. This is laborious and heavily dependent on both an available reference genome and correct gene annotations. Conversely, with adaptations of the original methodology, transposon libraries can be constructed and sequenced in roughly 1 week. Moreover, transposon libraries are annotation independent, meaning they can be constructed simultaneously with sequencing and annotation of new genomes, or updated as new annotations become available.

Despite its utility for the identification of essential genes on a whole-genome scale, some limitations exist. Benchmarking a TraDIS library with a gene deletion library in the same strain highlighted caveats of the method [3]. For TraDIS analyses, gene essentiality is inferred by quantification of transposon insertion density. As such, some genes with few transposon insertions may be falsely classified as essential. For example, some non-essential genes can only be disrupted by the transposon in an orientation that maintains the expression of downstream essential genes (termed ‘polar’ insertions). This produces half as many insertion events in the output data. Alternatively, DNA-binding proteins may prevent the transposon from inserting into the chromosome by physically occluding the transposon from the bound DNA, again resulting in fewer insertion events and genes being mislabelled as essential. Combining TraDIS screens with proteomic or Chip-Seq data can help identify these regions. Finally, some insertion events may produce a slow growth phenotype, resulting in mutants being less abundant within the total mutant pool; such mutants may also be misclassified as essential.

Relatedly, there is a scale of essentiality, which presents challenges to identifying a definitive ‘essential gene list’. For example, disruptions in some genes have immediate killing effects, such as interfering with ATP biosynthesis, DNA synthesis or ribosome assembly, while lethal insertions in genes involved in cell division or outer-membrane biosynthesis result in slower cell death [6]. The genomic background of the strain and growth conditions for each library impact which genes are essential. Moreover, libraries are constructed in artificial laboratory conditions, which are not representative of natural habitats. Conditional screens can identify genes essential for survival in different environments and stress situations, and it is preferable to work with the strain of interest where possible, rather than trying to draw conclusions from a laboratory or closely related strain, as genetic background strongly influences gene essentiality.

To address this gap, more recent studies have compared essential genes from multiple strains of the same species, or tested libraries in multiple environments. One study reports a minimum of four libraries, constructed in different strains of the same species, are needed to identify the core essential genome of a species [7]. Pan-genome or multi-conditional essential studies can be used to identify shared pathways or genes that are essential under all conditions versus those that are essential under specific growth conditions or in specific genetic backgrounds.

### Conditional screening

**Fig. 2.**
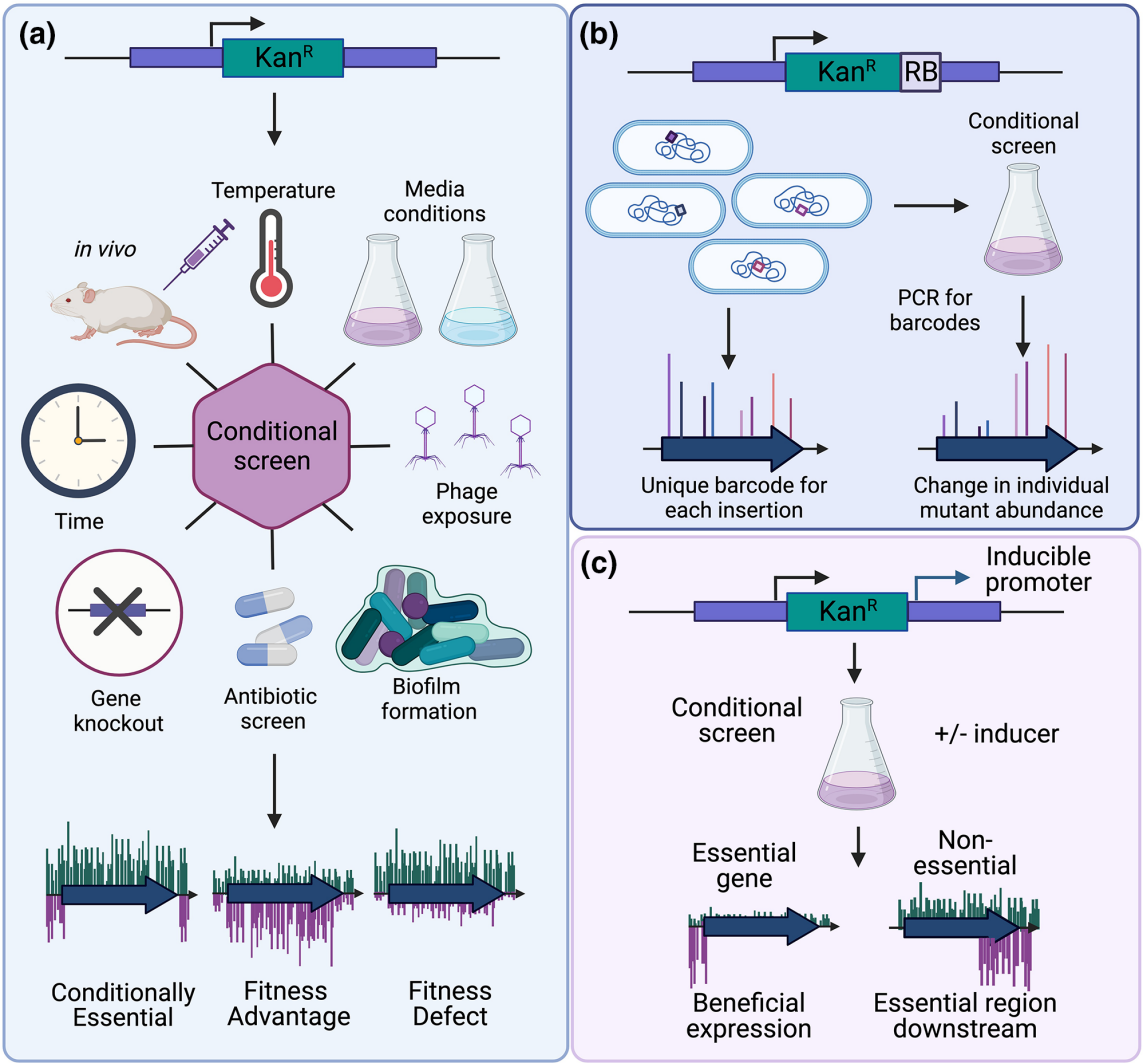
Applications of TraDIS. The applications of TraDIS. Traditional conditional screens are shown in (a), where an input library (green) is compared to the output library produced by growth in a condition (purple). Greatly reduced insertion density and frequency in a conditional mutant library corresponds to conditional essentiality of a gene or a fitness defect, while the inverse identifies genes where mutations are beneficial for survival. Two modifications of the transposon itself are shown. These include RB-TraDIS (b), where each transposon contains a unique random barcode that can be used to quickly identify the abundance of specific mutants under any conditions. Additionally, TraDIS-Xpress (c) uses an inducible outward facing promoter from the transposon to identify beneficial expression of essential genes under conditional screening and identify non-essential genes or regions where over-expression is similarly beneficial for survival.

As described, TraDIS can be applied to identify ‘conditionally essential’ genes [1]. A transposon mutant library can be grown under any number of conditions (Fig. 2a). Equivalent sequencing of the library before and after growth under selective conditions is used to measure the effect of a given mutation on fitness. Based on the differential abundance in the number of sequencing reads at each transposon mutation site, a fitness score is assigned to a given gene . This serves as a proxy for the change in number of those mutants between the input and output libraries, and their relative fitness in each condition. Mutants with greater fitness will have increased read depth compared to the input library, while genes that are essential or have a fitness defect in the condition will have significantly fewer or no reads in the conditional library. This allows for rapid, genome-wide assessment of phenotype-genotype relationships *en masse* in selective conditions. Indeed, numerous *in vitro* conditions have been queried through transposon library screening, ranging from temperature selection, media or nutrient requirement, biofilm formation, viable but non-culturable state entry, phage competition or resistance, motility screening, membrane permeability, antibiotic sensitivity and resistance mechanisms, and even the change in mutant abundance over time in long-term evolution experiments.

There are two main challenges with the application of TraDIS for infection studies: bottleneck effects and inoculum size. Bottleneck effects occur when stochastic events severely limit the size of the mutant pool. For instance, when an animal is given an oral inoculum of enteric pathogens it must first pass through the stomach before reaching the intestines. The population reduction from the acidic conditions constitutes a major bottleneck, and the cells that reach and colonize the intestines will not be representative of the initial population [8]. As a result, there is reduced genetic diversity among the surviving mutants and consequently not all mutants are assayed equivalently. If mutants without a fitness defect are lost stochastically, this can result in mislabelling of genes as conditionally essential. To limit bottleneck effects, inoculum size can be increased to account for downstream mutant loss or an alternative infection route can be used. However, this is not always feasible depending upon the infection model. For each organism and model system, only a certain number of cells are needed to cause disease (the infectious dose). If the number of mutants contained in a typical TraDIS library is larger than the lethal infectious dose, the library cannot be assayed in an animal. While the library complexity can be limited to address this, it increases the likelihood of stochastic effects. To address this, and adjust for statistical significance, the choice of inoculum size, infection route, and animal cohort size can be modified to avoid bottlenecks, maintain adequate library complexity, and produce sufficient output mutants for comparative study. Reducing the library complexity can decrease the number of animals required for appropriate mutant representation. However, low complexity transposon libraries have a lower number of unique insertion mutants, which reduces the ability to identify specific insertion points that may contribute to infection. Nevertheless, this maintains the ability to identify conditionally essential while avoiding bottleneck effects and minimizing the number of animals required.

### Genetic interaction

TraDIS can also be used to investigate the relationship between genes. Synthetic lethality refers to the phenomenon where the simultaneous loss of two genes leads to cell death, while the loss of either gene independently does not affect cell viability. A classic example of this is *mrcA* and *mrcB* in *E. coli*, which encode penicillin-binding proteins 1A and 1B, respectively, and are required for peptidoglycan biosynthesis. Conversely, using TraDIS it is also possible to identify genetic mutations that bypass gene essentiality, or synthetically viable gene pairs. For instance, *ftsH* is essential in the *E. coli* strain BW25113 genetic background. Functional FtsH degrades LpxC, which catalyses the first dedicated step in lipopolysaccharide (LPS) synthesis, and non-functional FtsH results in toxic accumulation of lipopolysaccharide (LPS) through unchecked activity of LpxC. However, in a TraDIS library constructed in a Δ*yhcB* strain, *ftsH* is not essential, indicating that high levels of LPS are not toxic to the cell in this genetic background and identifying *yhcB* and *ftsH* as a synthetically viable pair of genes [9]. Identifying synthetic pairings can point toward novel biochemical pathways, resistance mechanisms and the genetic context for genes of unknown function, which can be confirmed with other protein–protein interaction or gene expression data [2].

### Transposon modifications

As the scale of experiments with transposon mutant libraries increases, the economical and labour cost required for processing the resulting number of samples can be prohibitive. This largely stems from the multistep library preparation process prior to sequencing. To this end, random barcoding Tn-seq (RB-TnSeq) has been devised to screen a multitude of strains simultaneously under varying environmental conditions. RB-TnSeq utilizes unique barcode sequences as part of the transposon design (Fig. 2b). Following mutagenesis with the barcoded transposon, each mutant will contain a unique barcode, which can be matched to its respective insertion site during initial sequencing of the mutant library [10]. Subsequent screens using this mutant library can then be sequenced with a single step of PCR, and mutant abundances are tracked by comparing the abundance of each barcode. RB-TnSeq allows for mutants to be traced during growth in numerous conditions, which can aid in identifying putative functions for poorly characterized genes that become essential or have beneficial mutations [11]. Additionally, this technique can be used to study genes important for competition between bacteria, even between two mutant libraries simultaneously.

One often cited limitation of TraDIS is the inability to screen essential genes. However, with modification of the transposon, TraDIS can also be used to study gene overexpression and its effect on viability. The TraDIS-Xpress method employs an outward facing inducible promoter as part of the transposon (Fig. 2c). This provides a way to control the expression of genes downstream of the transposon insertion site. By inducing or repressing the promoter, it is possible to compare the effects of gene expression on survival. These screens can also identify multicopy suppressors, where a mutant over-expresses a particular gene such that it avoids an otherwise expected phenotype like antibiotic-induced killing. Recently, this method has been used to identify genetic factors that contribute to biofilm formation at various time points in the life cycle, and survival in several antibiotics [12].

### Analysis methods available

Several analysis methods are available depending on the transposon system used (detailed elsewhere: [1, 5]). Briefly, for the Tn*5* transposon, the publicly available BioTraDIS pipeline identifies essential genes using a bimodal distribution of insertion index scores (number of insertions normalized over the length of a gene) to set a threshold for essentiality. BioTraDIS and its companion AlbaTraDIS can be used for analysis of conditional essentiality and analysis of multiple library datasets to identify the effects on gene neighbourhoods. Recently, INSDENS has been introduced as an alternative to BioTraDIS, and avoids an arbitrarily set threshold for determining essentiality, which may be useful when comparing essential genes between different mutant libraries. AlbaTraDIS can be used to identify the impact of transposon insertion on gene neighbours to infer gene networks involved in conditional survival. Notably, all analysis outputs with TIS methodologies are annotation dependent.

In contrast, for Mariner *HimarI* systems, Essential Locus (EL) ARTIST and Conditional (Con) ARTIST pipelines identify essential genes and conditionally essential genes using a hidden Markov model. This sliding window approach is annotation independent and identifies genes and regions with significantly low insertion density. Currently, the BioTraDIS, INSDENS and AlbaTraDIS pipelines can be used on the User Interface (UI) Galaxy or run in unix environments. The ARTIST pipelines are only compatible with unix systems.

### Limitations of TraDIS

Construction of a mutant library is the first hurdle of transposon mutagenesis techniques. A major limitation of TraDIS is whether the species or strain of interest is genetically tractable. Even if the organism is amenable to genetic manipulation, there can still be further limitations for library construction: transformation capability of the strain with the transposome complex, or the plasmid vector, as well as growth parameters and time can significantly limit the number of mutants that are produced and the accuracy of the downstream analysis. However, there are now several different methods for the successful delivery and integration of a transposon, ranging from transformation of the transposome *in toto*, delivery of the transposon via suicide vectors, conjugation and phage-delivery. Following the successful construction of a library, other limitations arise such as the false-positive identification of essential genes (discussed above) and the inability to assay essential genes in downstream assays. Two complementary methods for the identification or screening of essential genes are CRISPRi and dub-seq. Briefly, CRISPRi involves silencing gene expression using a plasmid-encoded guide RNA to interfere with the expression of a target gene while dub-seq involves cloning chromosomal fragments into a plasmid and using the plasmid copy number as a means of altering gene expression level. While techniques like TraDIS-Xpress can address some of these issues, such as the screening of essential genes, the use of plasmid-based systems enables a more tightly controlled modulation of essential gene expression in phenotypic screens. Used together, these techniques can complement each other to provide a more complete picture of gene essentiality.

### Adaptations of TraDIS and future directions

Finally, the high-throughput nature and flexibility of TIS protocol lends itself well for further adaptation. This can be achieved through modification of the transposon itself or by coupling TIS with other high-throughput methods. The many applications of TIS methods have been reviewed extensively by Cain *et al*. [1]. Future directions for the use of TIS methods, will likely see an increase in multi-dimensional screens that integrate additional datasets and data types. However, there is scope for improving analysis of existing TraDIS data. A gap in TraDIS analyses is the availability of analytical tools that can interpret the more nuanced insertion profiles observed in an ultra-dense mini-Tn*5* library. There is a need for the hidden Markov model approach, currently available for Tn-seq analysis, that can predict essential regions independently of genome annotation within the context of surrounding data. While a sliding-window analysis option is available as part of Alba-TraDIS, the window size must be user-determined and does not incorporate wider genomic context. With the increase of machine-learning approaches for data analysis, we look forward to new tools with greater and more accurate predictive power for essential gene analysis.
